# The Viscoelastic Properties of Passive Eye Muscle in Primates. III: Force Elicited by Natural Elongations

**DOI:** 10.1371/journal.pone.0009595

**Published:** 2010-03-08

**Authors:** Christian Quaia, Howard S. Ying, Lance M. Optican

**Affiliations:** 1 Laboratory of Sensorimotor Research, National Eye Institute, Bethesda, Maryland, United States of America; 2 Department of Ophthalmology, Wilmer Eye Institute, The Johns Hopkins Hospital, Baltimore, Maryland, United States of America; University of Arizona, United States of America

## Abstract

We have recently shown that in monkey passive extraocular muscles the force induced by a stretch does not depend on the entire length history, but to a great extent is only a function of the last elongation applied. This led us to conclude that Fung's quasi-linear viscoelastic (QLV) model, and more general nonlinear models based on a single convolution integral, cannot faithfully mimic passive eye muscles. Here we present additional data about the mechanical properties of passive eye muscles in deeply anesthetized monkeys. We show that, in addition to the aforementioned failures, previous models also grossly overestimate the force exerted by passive eye muscles during smooth elongations similar to those experienced during normal eye movements. Importantly, we also show that the force exerted by a muscle following an elongation is largely independent of the elongation itself, and it is mostly determined by the final muscle length. These additional findings conclusively rule out the use of classical viscoelastic models to mimic the mechanical properties of passive eye muscles. We describe here a new model that extends previous ones using principles derived from research on thixotropic materials. This model is able to account reasonably well for our data, and could thus be incorporated into models of the eye plant.

## Introduction

Mathematical models of muscles usually ignore the dynamic properties of *passive* muscle tissues, and focus instead on the *active* properties. Accordingly, the length-tension-innervation relationship and the force-velocity relationship have received most of the attention. In skeletal muscles this approach is easily justified, because in natural conditions they operate in a length range over which passive muscle forces are negligible. It has been known for a long time [1] that the situation is vastly different in extraocular muscles, as passive forces are significant well within the physiologic eye position range (the so-called oculomotor range). In humans for example, when the eye is deviated by just 10° from straight-ahead, passive tissues already contribute 50% of the static force exerted by the antagonist muscle. This fraction increases quickly with eccentricity [2], and there is every reason to believe that dynamic forces are similarly large [1]. Despite their importance, the dynamic properties of passive eye muscles have not been systematically measured.

To fill this experimental gap, in the first paper in this series [3] we quantified the dynamic forces elicited by small step-wise elongations applied to passive extraocular muscles in monkeys (measured *in vivo*). We found that the peak forces are indeed quite large, that the force can still be significant one second after the end of the elongation, and that it tapers off slowly for a long time after that. On average, it takes 4 s for the dynamic force to decay to 10% of its peak value. In the second paper in this series [4] we attempted to fit standard nonlinear viscoelastic models to our data, focusing in particular on Fung's quasi-linear viscoelastic (QLV) theory [5], [6]. Under this theory, the nonlinear viscoelastic process that produces a strain given a stress is interpreted as the cascade of a static nonlinearity followed by a set of linear processes, whose outputs are summed together. We found that Fung's original model could reproduce reasonably well the single-step data, but its most recent extension, the so-called AQLV model [7], could do even better. This model is more flexible, since it does not constrain the post-elongation decay to be independent of length. However, both models failed to reproduce sequences of two steps separated by a short time interval. We showed that this failure was due to the structure of the models, and hence could not be overcome by adjusting their parameters.

Designing a model capable of reproducing the double-step data is certainly a worthwhile effort *per se*. However, our main scientific interest is to build a model of the eye plant able to reproduce the eye movement deficits observed after muscle paralysis [8]. We are thus mostly interested in the passive forces that are generated in eye muscles during typical eye movements, what we call “natural elongations”. Accordingly, in this paper we describe two new sets of experiments on passive extraocular muscles in anesthetized and paralyzed monkeys. In one set of experiments we imposed on the muscles elongation profiles that are similar to those experienced by the antagonist muscle during saccadic eye movements (and the fast phases of the vestibulo-ocular reflex). In another set of experiments we continuously stretched the muscles at a constant speed, simulating the elongations experienced during smooth pursuit (and the slow phases of the vestibulo-ocular reflex). These new data sets directly estimate the force exerted by paretic eye muscles during rotations of the eye in their off direction (i.e., in the direction in which the muscle normally acts as an antagonist).

Besides reporting our measurements on natural elongations, here we compare the force measured with the force predicted by the models that we previously used [4] to fit the (single) step data. The reader might wonder why we used models that we have already shown to be inappropriate to fit sequences of steps. There are actually two reasons. First, viscoelastic responses are quite complex, and without a model that acts as a reference it is often difficult to have an idea about what force to expect under an elongation pattern given the response to another elongation pattern. The models provide us with a sense of what is an “expected” response. The second reason is to test a speculation made by Pipkin and Rogers [9]. They proposed a method to find a non-parametric model capable of reproducing the viscoelastic properties of nonlinear materials. They suggested that the force elicited by elongation steps could be used to find a first order approximation of the system under study. Sequences of two steps could then be used to refine this approximation by adding a second term to the model, and so on. This successive approximation approach is standard in non-parametric, nonlinear, system-identification (e.g., Volterra and Wiener series [10]). Based on experimental measurements on man-made materials (mostly plastics and polymers), they also noted: “We are convinced that experiments involving continuously variable loading are poor tests of the extrapolation from step data, because the extrapolation makes such accurate predictions for such experiments that nothing new is learned” (Pg. 70). If this observation also applies to biological materials, the models we previously described might provide a first order approximation for the type of elongation patterns that we are interested in simulating.

As we will show, the models did not pass this test, implying that Pipkin and Roger's assertion cannot be assumed to apply to biological materials. Interestingly, we found that the force exerted by a muscle following any elongation is largely independent of the elongation speed profile or amplitude, and it is almost entirely determined by the final muscle length. Coupled with our previous finding that in a sequence of elongations only the last one determines the force, this implies that the final muscle length by itself largely determines the post-elongation decay of the force, regardless (within limits) of the muscle's length history. As far as we know, this type of behavior has not been previously described in either biological or man-made materials. Accordingly, even though there are very general nonlinear viscoelastic theories that yield models that can account for any stress-strain relationship, we could not find any published viscoelastic model able to accommodate this post-elongation behavior. A careful comparison of the predictions of the QLV [5] and AQLV [7] models with the data we recorded led us to formulate a new model, which uses principles derived from the study of thixotropic materials to extend those previous models. We show that this relatively simple extension yields reasonably good fits to all the forces we measured, making it a good candidate for inclusion in a model of the eye plant.

## Methods

The methods used to collect the data presented in this paper have been described in great detail in the previous papers in this series [3], [4]. Here, only a brief summary is provided.

### Ethics Statement

All procedures were in agreement with the USA Public Health Service policy on the humane care and use of laboratory animals. All protocols were approved by the Animal Care and Use Committee of the National Eye Institute. All procedures were non-recovery, and were carried out under deep anesthesia. Accordingly, the experiments introduced no suffering beyond that attributable to the injection of the inducing anesthetic. As mandated by the aforementioned policy and committee, welfare of the animals during their stay at our primate facility was promoted by pair housing animals, providing regular access to a large shared play room, and providing a variety of objects in their home cage, specifically chosen for the purpose of enrichment.

### Animals

Eye muscle forces were measured in three adult rhesus monkeys (*Macaca mulatta*), ranging in weight from 8 to 14 Kg (identified as m2, m3, and m4). None of the animals had been previously used in any experiment, and their eyes and orbits were thus pristine.

### Surgical Procedure

The animal was placed supine on the surgical table, intubated, ventilated and anesthetized with isoflurane (2–4%) in oxygen. Paralysis was induced with pancuronium bromide (0.05–0.10 mg/Kg IV), and was maintained by administering a reduced dose (0.025–0.050 mg/Kg IV) every 45 minutes until the end of the procedure. The paralytic agent was used to ensure that the muscles were completely passive. At the end of the procedure, and while still deeply anesthetized, the animal was euthanized with an overdose of sodium pentobarbital (150–250 mg/Kg IV).

### Experimental Procedure

After the animal had been anesthetized, its head was fixed, looking straight up, in a stereotaxic device. The conjunctiva was then incised in correspondence with an eye muscle insertion on the globe, and the muscle tendon was connected to the measuring apparatus. The techniques and materials used to perform this connection are described in great detail elsewhere [4]. The muscle force was measured using an Aurora Scientific (Aurora, ON, Canada) 305C Dual-Mode Muscle Lever System. In the experiments described here we always imposed the muscle length, and measured the corresponding change in force (the SI standard unit of force is the newton (N), but muscle force is traditionally measured in units of *gram force*: 1 gf≈0.0098 N). The input/output analog signals from this device were generated and acquired through an A/D-D/A interface board (National Instruments, NI USB-6211) connected to a laptop PC and controlled by LabView (National Instruments, Austin, TX). The experiment was controlled by a custom Java program that communicated with LabView, displayed the data in real-time, and stored it for later analysis.

The forces reported in this paper were elicited by imposing the following elongation patterns:

- Saccade-like elongations (i.e., half-sinusoid velocity profiles), having a range of amplitudes (between 1 and 4 mm), peak speeds (between 60 and 160 mm/s) and starting from different initial muscle lengths.
- Constant-speed stretches spanning the entire elongation range, at various speeds (0.1, 1, 10, 80, and 160 mm/s).
- Sequences of double saccade-like elongations, separated by variable time intervals (0.01, 0.1, 1, and 45s), from initial lengths spanning the entire elongation range.

Only lengthening was tested, because it was technically not possible for us to measure the forces during shortening (they become negative for even relatively low shortening speeds). Double-saccades were added toward the end of our experiment, and so all the data for this condition comes from two muscles in one monkey. Note that in all our experiments the speed and acceleration applied to the muscles were chosen so that they never exceeded those experienced by the muscles under normal behavioral conditions.

The elongation range was determined separately for each muscle. The range tested always covered the entire oculomotor range (i.e., the set of lengths that are achieved in physiologic conditions, which in monkeys correspond to approximately ±45° of rotation), but never exceeded it by more than one mm. Accordingly, the elongation range tested was always about eight mm. Before recording we preconditioned the muscles by repeatedly (5–10 times) stretching and releasing them sinusoidally over their entire range (which is standard procedure in tissue rheology to guarantee repeatable results; the relatively low number of cycles used here is justified by the *in vivo* condition we used). For all muscles tested, we ran a block of three-four ramps at the beginning and at the end of the experiment to test for any possible deterioration of the muscle. We never observed any significant change in these test trials.

In our experimental preparation, the raw force measures are affected by a significant heartbeat and respiration-related noise. As explained at length in the first paper in this series [3], we devised a method to very effectively remove, *post hoc*, both of these noise components. The residual measurement noise was extremely small, at or below the level of our instrumentation accuracy.

### The QLV Model

Rather than just showing the time course of the force elicited by our experimental paradigm, which by itself cannot be easily put in context, we present here the data we collected from monkey extraocular muscles together with the output of model simulations. The first model we use, described in the previous paper in this series [4], is the QLV model proposed by Fung [5], [6], slightly reformulated (compare with Eq. 16 in [4]) for reasons that will become clear later on[4]:
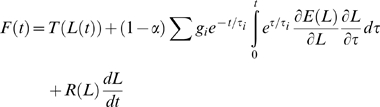
(1)The model has 15 degrees of freedom (DOF). The first term in Eq. 1 is the length-tension relationship; it quantifies the elastic force, and it is a function of length (4 DOF). It is an estimate of the force exerted by the muscle when a length L is maintained for a very long time (i.e., at equilibrium). The second term in Eq. 1 represents what we call the purely viscoelastic force, and it is a functional of the speed of elongation. It is actually the sum of the force generated by seven separate processes, each characterized by a time constant τ_i_. The number of processes and the value of the time constants were selected based on the recording window and the noise level [3], and are thus not DOF of the model (they were not fit to the data). The moduli g*_i_* and α are thus the 8 DOF of this part of the of model. The third term in Eq. 1 represents a pure viscous force, and it is a product of a length-dependent viscosity (3 DOF) and the speed of elongation. As noted in the previous paper, we have reasons to believe that this last term is in fact due to an artifact, and should not be considered part of the muscle model. The only difference between this model and the original QLV model is the addition of this term.

### The AQLV Model

The second model we use, also described previously [4], is based on the AQLV model proposed by Nekouzadeh and colleagues [7]. The model (Eq. 20 in [4]) has 35 degrees of freedom (DOF), and its equation is:
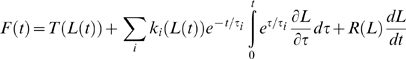
(2)The first term in Eq. 2 is again the length-tension relationship (4 DOF). The second term is the purely viscoelastic force, and it is a functional of the speed of elongation. Just like for the QLV model, it is the sum of the force generated by seven separate processes, each characterized by a time constant τ_i_. The stiffness *k_i_* of each process is a function of length (4 DOF for each process). The third term in Eq. 2 is the viscous force (function of length, 3 DOF). The only difference between this model and the original AQLV model is the addition of this last term.

### Simulations

The models presented in this article were simulated numerically in Python (using the freely available packages weave, numpy, scipy, and matplotlib). The scripts are available upon request. Parameter optimization was carried out using a commercial optimization package (modeFRONTIER™, Esteco s.r.l., Trieste, Italy).

## Results

### Force during Saccadic Elongations

We imposed on passive extraocular muscles (EOMs) large elongations characterized by a half-sinusoidal velocity profile. The amplitude of the elongations ranged between 1 and 4 mm, and the peak speed varied between 60 and 160 mm/s. These patterns of elongation are very similar to those experienced by the antagonist eye muscle when a saccadic eye movement is executed (given the radius of a monkey eye, they correspond to saccadic amplitudes between 6° and 25°, and peak velocities between 360°/s and 1000°/s), and we thus refer to them as saccadic elongations. For each elongation, speed and amplitude were selected to fall more or less on the “main sequence” for saccades [11], [12], and are thus positively correlated. In the first monkey we also compared saccadic elongations having the same amplitude but different peak velocities; since this condition did not yield particularly interesting results, we dropped it in the other animals.

**Figure 1:**
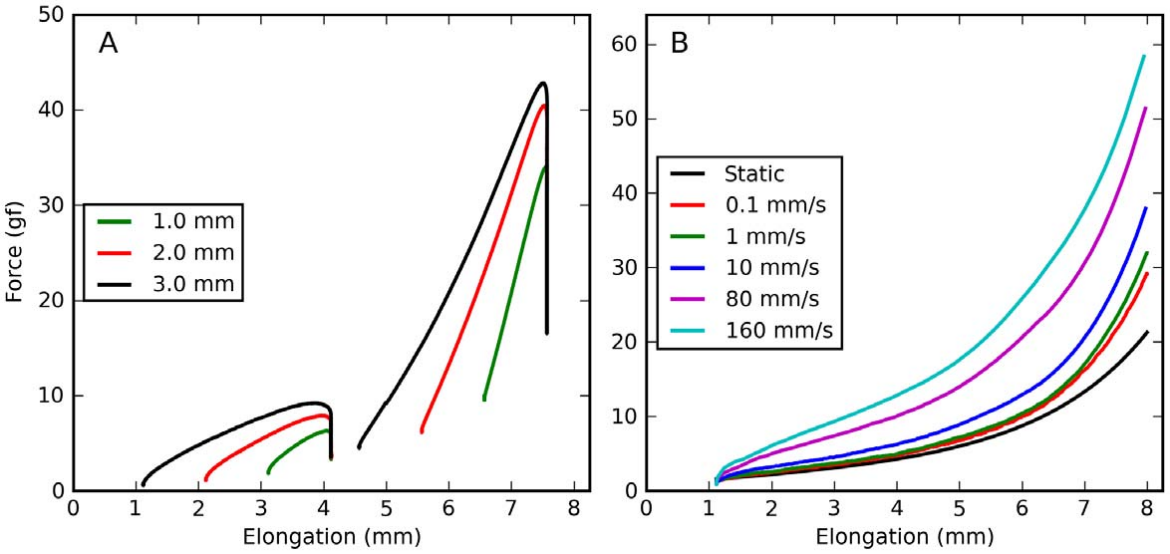
Force during natural elongations. This study focuses on the forces generated by passive eye muscles when subjected to elongations similar to those experienced under physiologic conditions. We used elongations characterized by a half-sinusoidal velocity profile (like that induced by saccadic eye movements), as well as constant-speed stretches. **A**: Forces induced by six different saccadic elongations. Elongations of 1.0 mm (green), 2.0 mm (red), and 3.0 mm (black) are shown, ending either at an intermediate length or near the limit of the elongation range tested. The force is plotted as a function of the instantaneous elongation. **B**: Forces induced by constant-velocity stretches, at different speeds and covering most of the elongation range. Black: Static length-tension relationship. Because the elongation was not terminated abruptly, but rather decelerated smoothly, the force started to drop a few ms before the actual end of the elongation. For clarity, this section of the force record is omitted from the figure. All the data comes from the same muscle (LR in m4).

In Fig. 1A we report the force generated under six such elongations, all in the same muscle (the lateral rectus from m4). We show elongations of three amplitudes, and for each amplitude we used two different initial lengths. The force is plotted as a function of muscle length. Comparing elongations that have different amplitudes but the same final length, we see that the force increases with the amplitude of the elongation. Comparing elongations that have the same amplitude, but start from a different initial length, we see that the force increases with the starting length. Qualitatively speaking, this is what would be expected from a nonlinear viscoelastic system. From a quantitative point of view, the only point we would like to stress at this time is that these forces can be quite large. For example, an 18° saccade starting from 6° of eccentricity can be expected to induce 10 gf of passive force in the antagonist EOM (approximately 6 gf above the resting level force for the final elongation). Given the forces at play in the oculomotor system, passive forces are indeed far from negligible under natural conditions.

### Force during Constant-Speed Elongations

We also measured the force generated when constant-velocity stretches covering most of the length range are applied. Throughout our experiments, the forces induced by these elongations were used to verify whether there had been any tissue deterioration during the experimental session (they were the first and last sets of elongations applied). However, they also provide important additional information about the viscoelastic properties of muscle. In the first two monkeys, we tested speeds of 1 mm/s, 10 mm/s, 80 mm/s, and 160 mm/s. In the last monkey we also imposed a slower stretch, with a 0.1 mm/s speed. Since the elongation range was about 8 mm in all animals, the duration of the stretches ranged between 50 ms and 80 s.

In Fig. 1B we report the data from a full set of constant-speed elongations in one muscle (once again the lateral rectus from m4; all other muscles tested exhibited the same behavior). The force produced during each elongation is plotted as a function of the instantaneous length; the static force, extrapolated from the relaxation response to the step elongations [3], is also plotted. Not surprisingly, the higher the speed the larger the force. This is very similar to what was found by Collins, over a more restricted range of speeds, in the cat passive lateral rectus [13] (his Fig. 8). Our data contradicts, however, two of Collins observations. He suggested (Pg. 290) that a stretch performed at 0.2 mm/s can be used as a direct estimate of the static force. However, our data reveals that stretches at even lower speeds (red trace in Fig. 1B) can induce a considerable dynamic force. Because of the slow processes we have previously described, with time constants of 40 seconds or more, this behavior should not be considered surprising, and it is in fact predicted by the various viscoelastic models that we have used to simulate these elongations (shown below).

The second conclusion that Collins drew from his data is that what he termed the “viscosity” of the muscle decreases with speed (his Fig. 24). To understand what he meant it is useful to define as “dynamic force” the difference between the force measured during a constant-speed stretch and the static force at the same length. Collins observed that, for example, the dynamic force generated during a 100 mm/s stretch is much smaller than 10 times the dynamic force generated during a 10 mm/s stretch. If the stretches were applied to a system consisting of an elastic element in parallel with a viscous element (a so-called Voigt element), the dynamic force divided by the speed would indeed provide an estimate of the viscosity of the system. However, viscoelastic systems like the one we are studying are akin to a set of Maxwell elements (the series connection of an elastic and a viscous element) connected in parallel. In such models the dynamic force cannot be attributed to a single viscous element, but it is instead distributed across a set of different processes, characterized by different time scales. Because the stretches tested have different durations, covering the range of time constants of these processes, they drive the processes differently. Accordingly, the relationship between the dynamic force and the speed of the stretch says next to nothing about the “viscosity” of the system. As we will show below, the QLV model exhibits this same behavior without having to invoke the shear thinning effect suggested by Collins [13].

### Relaxation after Saccadic Elongations

**Figure 2:**
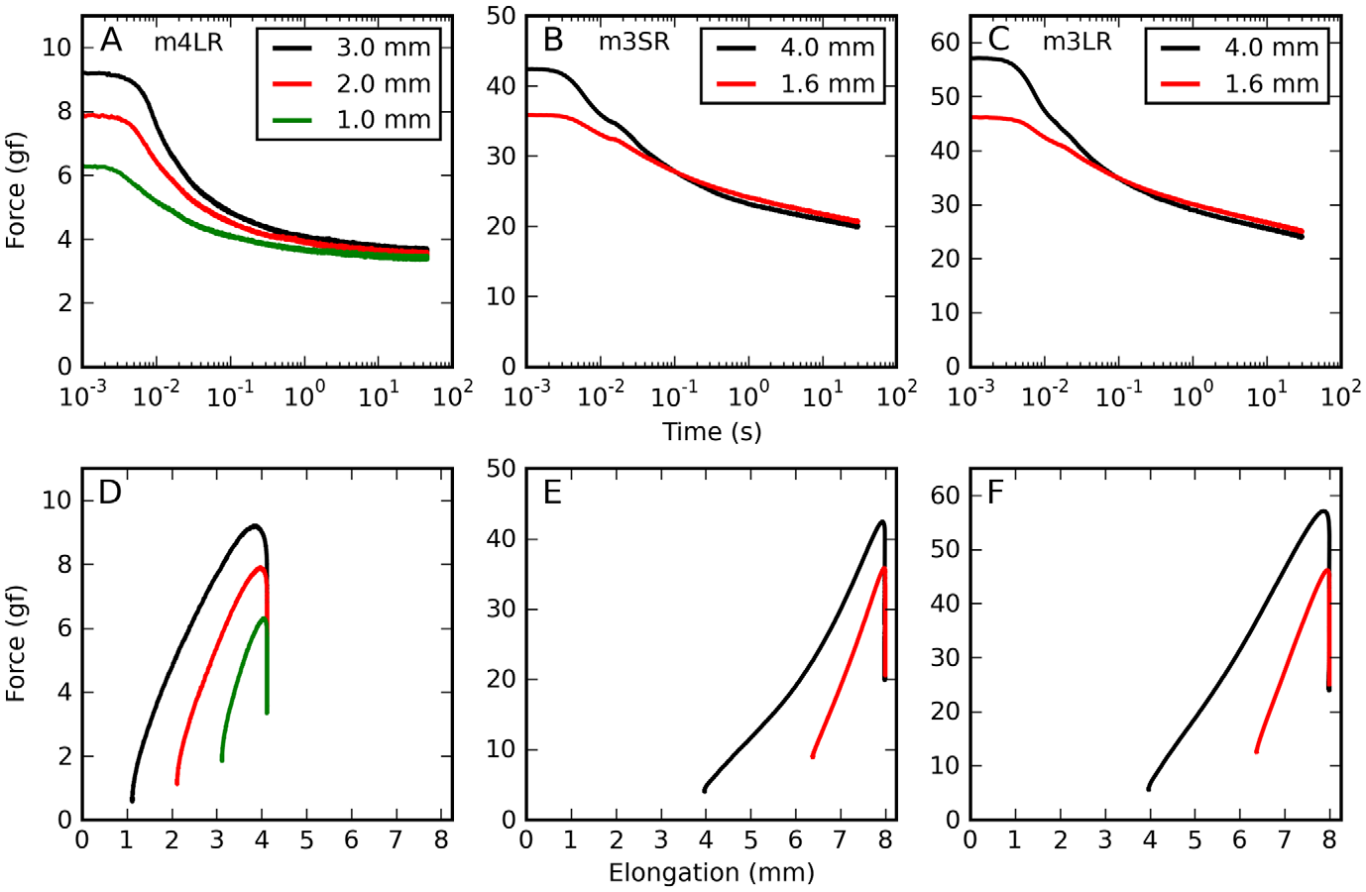
Force relaxation after saccadic elongations. Forces induced by saccadic elongations characterized by different amplitudes but with a common final elongation. **A**: 1 (green), 2 (red), and 3 (black) mm elongations terminating at an intermediate elongation, applied to the lateral rectus in m4. Note how the decay phase is almost independent of the elongation after 100 ms. **B**: 1.6 (red) and 4 (black) mm elongations terminating at the largest elongation tested, applied to the superior rectus in m3. Note how during the decay phase the force for the larger elongation becomes lower than that for the shorter elongation (the traces cross-over). **C**: Same as B, but in a different muscle (lateral rectus in m3). Also in this case the force crosses-over. **D–F**: Here we show, for the same elongations represented in the top row, the force induced in the muscle as a function of length.

Since vision is severely impaired when the eyes move, saccadic eye movements must not only be performed quickly, but must also come to an abrupt end. Hence, the forces at play after an eye movement are just as important as those occurring during the movement. Given our previous reports, it is to be expected that after saccadic elongations the force will decrease over a long time, following a multi-exponential (or power-law) time course. To get a quantitative idea of how the kinematics of an elongation affect the time course of the decay, we compared saccadic elongations with different amplitudes and velocities, but all ending at the same muscle length. Overall we collected six such sets of elongations, with two or three elongations in each set; as noted above, larger amplitudes were associated with larger peak speeds. What we found is not in line with the behavior of traditional viscoelastic models. As the amplitude and the speed increase, the force during the elongation also increases, as expected (Fig. 2, bottom row). However, after the elongation phase ends the force drops faster for the larger/faster elongations than for the smaller/slower ones, so that there is very little difference throughout most of the decay phase (Fig. 2, top row). After about 100 ms the difference between traces is very small, a fraction of one gf. Note that for large final elongations (panels B and C) there is actually a small cross-over, so that larger elongations are associated with smaller relaxation forces.

It thus appears that shortly after a saccadic elongation, the force decorrelates from the elongation speed and amplitude, and converges to a common value that is mostly determined by the current muscle length and by the time elapsed since the end of the elongation.

### Relaxation after Constant-Speed Elongations

**Figure 3:**
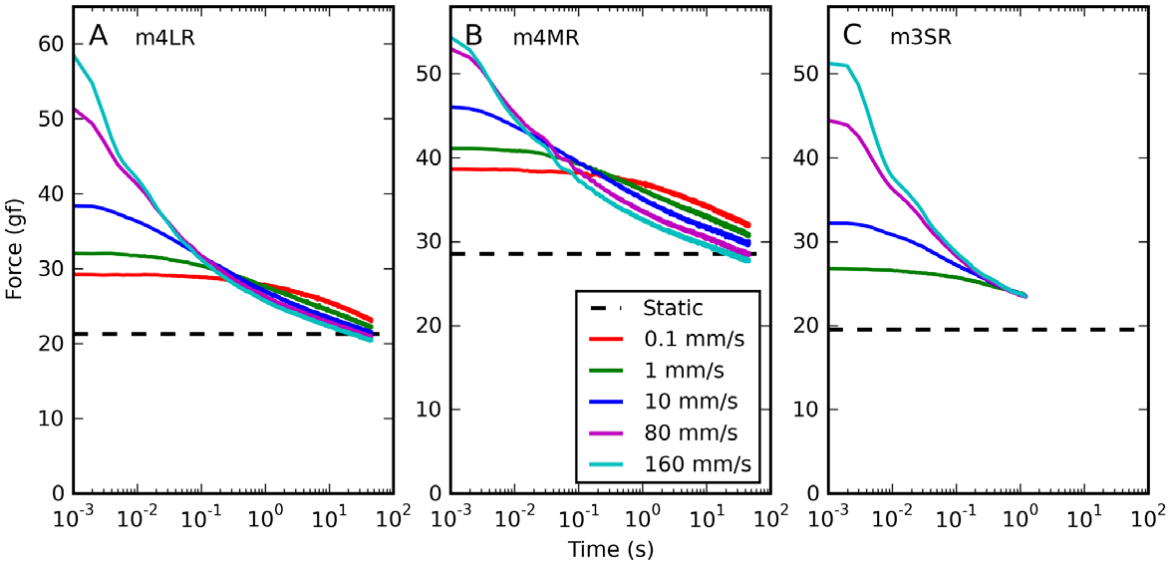
Force relaxation after constant-speed elongations. Time course of the force decay after the completion of constant-velocity stretches. Dashed black line: Static force predicted by the length-tension relationship. Different colors indicate different stretch speeds (see key). Each panel contains data from a different muscle. Note the cross-over between 20 ms and 1 s after the end of the stretch. Before the cross-over higher stretching rates are associated with higher forces, but after the cross-over higher stretching rates are associated with lower forces. Only in the last two muscles (shown in A and B) do we have data long after the end of the stretch. But even when the record is short (panel C) the pattern is evident.

We also measured the force decay following constant-speed elongations. In this case all of our elongations have a common final muscle length, and thus we could directly compare all the constant-speed elongations, separately for each muscle. We again found (Fig. 3) that the force dropped faster after fast elongations than after slow elongations. Even though the peak forces were considerably different, within about 100 ms all the traces come together. In this case the cross-over that we observed in some saccadic elongations was pervasive and of a much larger magnitude: in all cases, the higher the force at the end of the elongation, the lower the force 1 s later. In the first two monkeys we only recorded a short period after the end of the elongation (Fig. 3C), and so we have long relaxation responses only in two muscles (both shown). Nonetheless the pattern described is consistent across all muscles tested.

### Simulations: Current Models

As we noted in the Introduction, the complexity of nonlinear viscoelastic systems defies most people's intuition. Consequently, although a careful inspection of the data is useful, having a computational model as a reference for what to expect is necessary for a deeper understanding. Accordingly, we will now use two models to simulate the same elongations that we just described. The first model is Fung's original QLV model [5], slightly modified to account for the purely viscous component we observed in our measurements (see Methods, Eq. 1). The model parameters are different for each muscle, and are listed in Table 2 in our previous paper [4]. The second model we use is the AQLV model proposed by Nekouzadeh and colleagues [7], again slightly modified (see Methods, Eq. 2). Also in this case the model parameters are different for each muscle, and are listed in Tables 3–6 in our previous paper [4]. For both models the parameters were obtained fitting the response to 0.5 mm long step-wise elongations, which are reproduced quite well by both models; the QLV model used here is actually an average fit over the length of the muscle, and so it does not fit the step data as well as the AQLV model. However, the QLV has fewer parameters (15) than the AQLV does (35).

**Figure 4:**
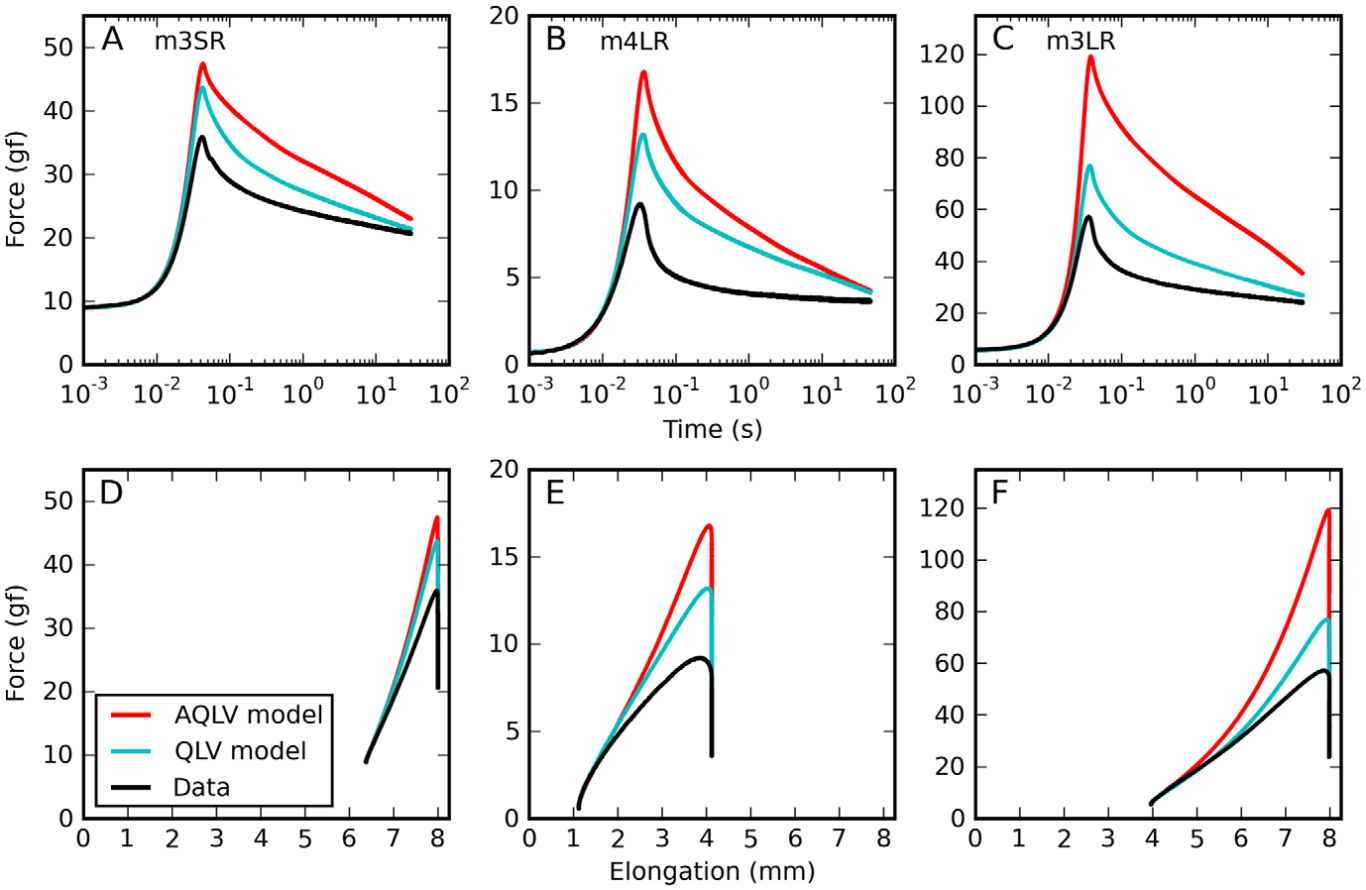
Saccadic elongations: model predictions. Forces induced by saccadic elongations. Data (black), and force predicted by the AQLV (red) and QLV (cyan) models, which in our previous paper we fit to the step-wise elongations. **A**: Short elongation (1.6 mm) starting from a large initial elongation, applied to the superior rectus in m3. **B**: Intermediate elongation (3.0 mm) starting from a small initial elongation, applied to the lateral rectus in m4. **C**: Large elongation (4.0 mm) starting from an intermediate initial elongation, applied to the lateral rectus in m3. **D–F**: Here we show, for the same elongations represented in the top row, the force induced in the muscle as a function of length. Note how in all cases the model fits the data well for the first 0.5 mm of the elongations (the step size to which the parameters were fit), and then increasingly overestimates the force. This overestimate lasts throughout the relaxation phase.

For all saccadic elongations tested (22 over five muscles), both models grossly overestimate the force exerted by the muscle, the AQLV more so than the QLV (three representative examples are shown in Fig. 4). [4] The discrepancy ensues shortly after the start of the elongation, and lasts throughout the entire relaxation phase. When the data and the simulations are plotted in the force-elongation plane (bottom row in Fig. 4) it appears clear that the models' output matches the data only for the first 0.5 mm or so, and then they diverge. We assume that there is nothing special about this distance, since that was the length of our steps, to which the models were fit. This data is thus consistent with our previous observation that, for sequences of two steps, both models fit well the force induced by the first step but during (and following) the second step predict a force larger than that actually measured in muscle, and the AQLV more so [4].

**Figure 5:**
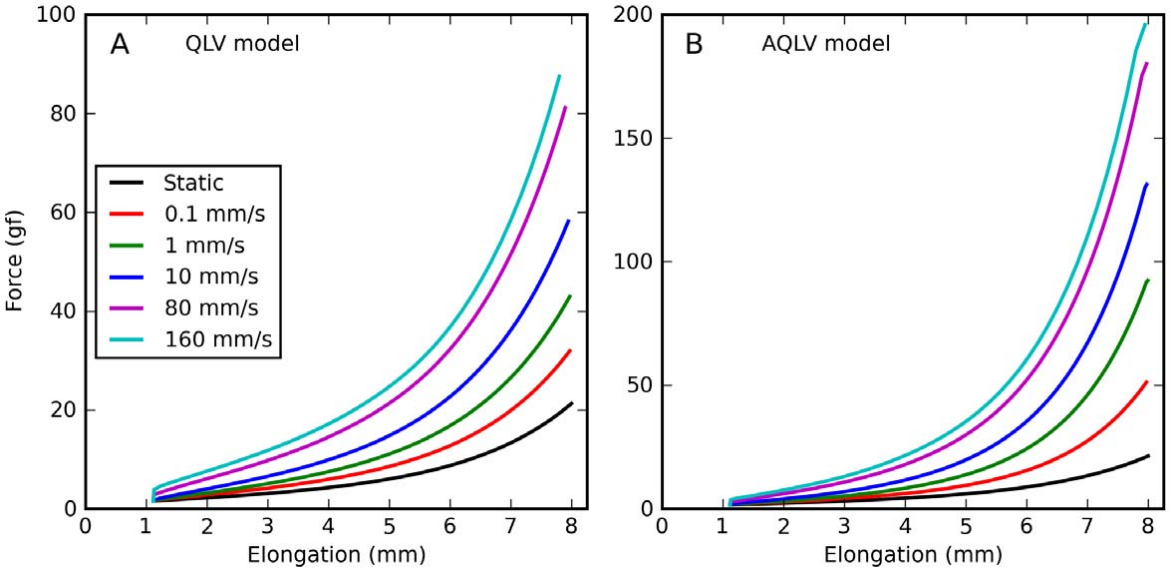
Constant-speed elongations: model predictions. Here we simulated a set of constant-speed elongations using the QLV and AQLV models, using the parameters derived in our previous paper by fitting the small-step data recorded from the lateral rectus in m4. These simulations should be compared with the data in Fig. 1B. Qualitatively the forces actually measured and those simulated are quite similar. Note in particular that even very slow elongations can result in forces considerably higher than the static force, and that the force grows less than proportionally to the elongation speed. However, both models grossly overestimate the force, and the AQLV more so (note the different ordinate scales).

We obtained a very similar result when we simulated constant-speed elongations. In Fig. 5 we report simulations of the same elongations described in Fig. 1B. Clearly, both the QLV model (Fig. 5A) and, to an even large extent (note different scales on the ordinates), the AQLV model (Fig. 5B) dramatically overestimate the force measured (cf. Fig. 1B). This discrepancy increases (in absolute terms) with the speed of the elongation. However, the models exhibit some of the same qualitative features observed in the data. First of all, even for extremely slow elongations the force predicted is considerably higher than the steady-state force. Furthermore, the dynamic force grows less than proportionally to the elongation speed (even though neither model contains any shear thinning effect).

**Figure 6:**
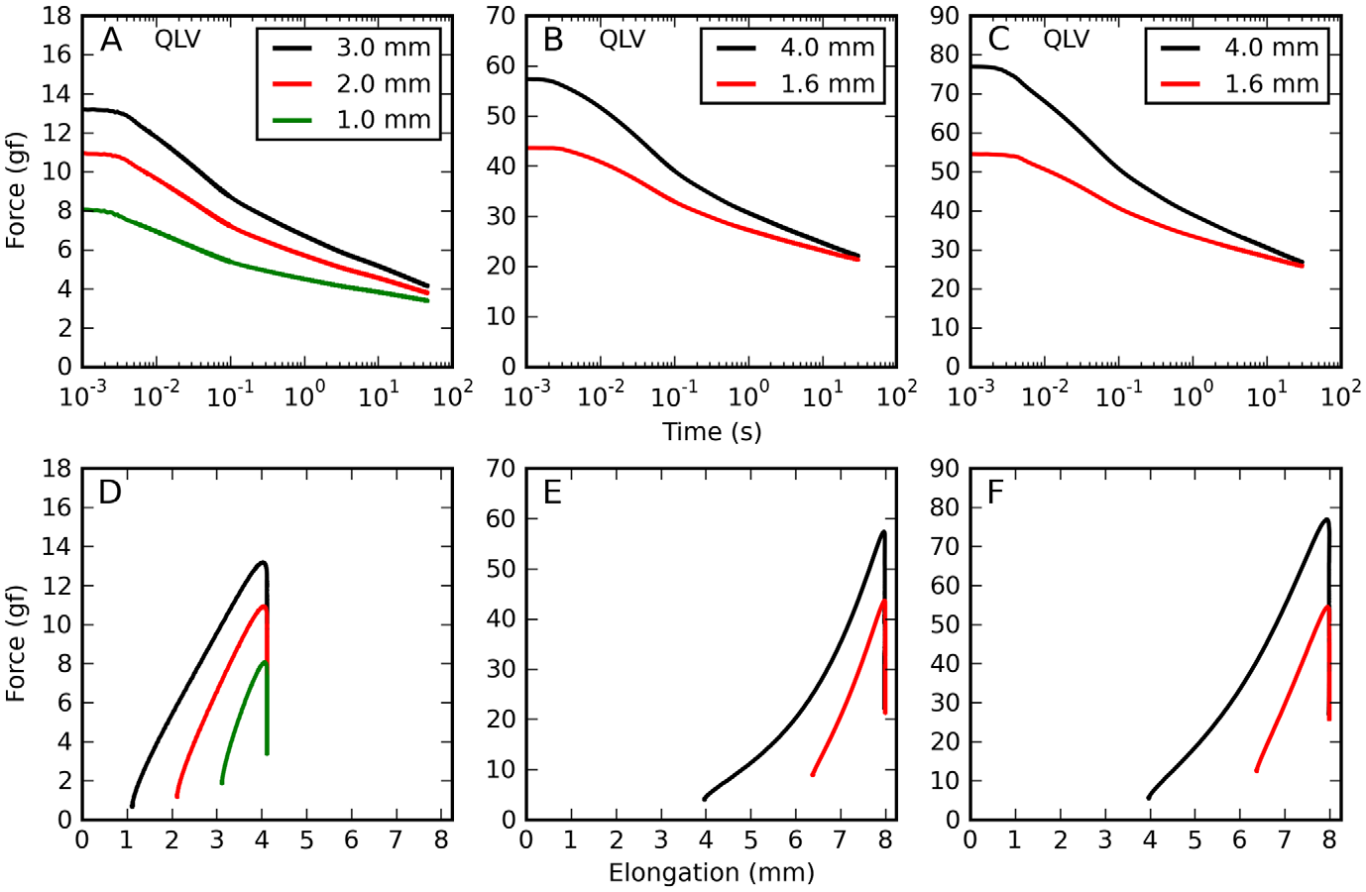
Force relaxation after saccadic elongations: model predictions. Simulations of the elongations described in Fig. 2 using the QLV model. **A–C**: Simulations of the force decay after elongations having different amplitudes (and speed) but the same final length. **D–F**: For the same simulated elongations, the force is plotted as a function of elongation. Larger elongations give rise to larger forces, as seen in the data. However, in the simulations this difference persists throughout the decay phase: the traces do not come together quickly, and there is no cross-over. Unlike what was seen in the data, the simulated forces are different not only during the elongation (bottom row), but also throughout the relaxation phase (top row).

We next simulated the same saccadic elongations that we described in Fig. 2, i.e., sets of elongations having different amplitude and speed, but terminating at the same length. In Fig. 6 we plot the predictions of the QLV model (note that there is no data in this figure, only simulation results). During the elongation (bottom row), the simulations appear to be simply a scaled up version of the data, which is not particularly surprising given our previous simulations (Fig. 4). The behavior during the decay phase is however qualitatively different: in the simulations the larger peak forces are carried over to the decay phase, and the force difference between the various elongations shrinks slowly. Unlike what we observed in muscles, the relaxations do not quickly converge, and there is never any force cross-over. With the AQLV model (not shown) the differences are quantitatively even more dramatic, but from a qualitative standpoint the AQLV behaves just like the QLV model.

We also measured saccadic elongations having the same amplitude and starting length, but different peak speeds. The speed differences we used were not very large: 60 mm/s vs. 100 mm/s for 1.6mm elongations and 100 mm/s vs. 160 mm/s for 4 mm elongations. When we simulated these elongations, both models predicted small peak force differences, and convergence between the traces within 100 ms. This was also observed in the data, but since it is not very informative we are not showing it.

Finally, we used the models to simulate constant-speed elongations. In the muscle we observed quick convergence of the relaxation responses, and extensive cross-over of the traces (Fig. 3).

**Figure 7:**
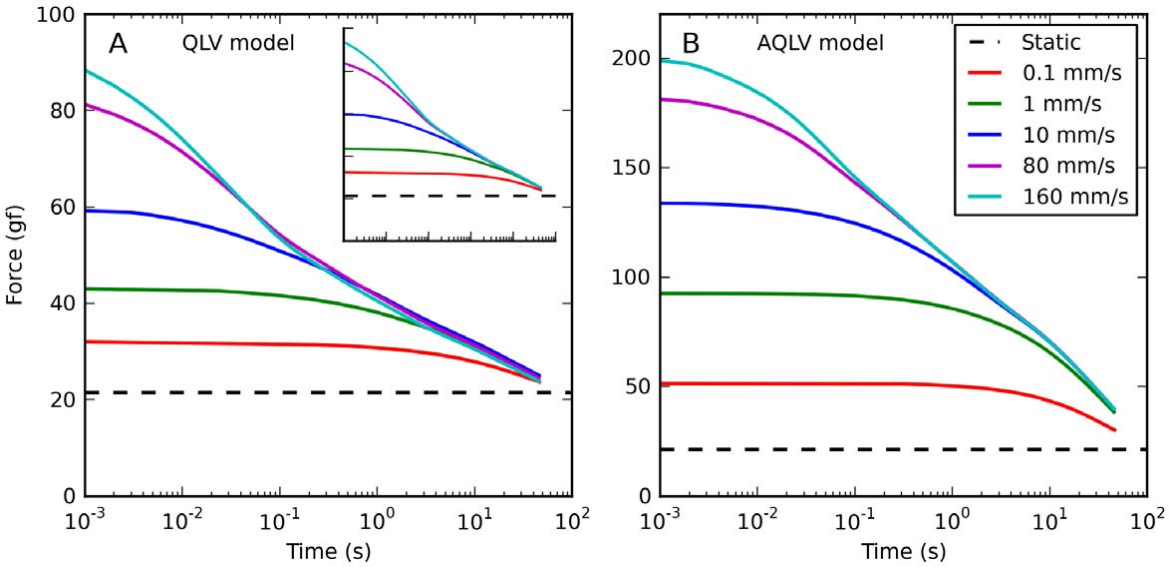
Force relaxation after constant-speed elongations: model predictions. Simulations of the same elongations described in Fig. 3A using the QLV and AQLV models. The extensive cross-over observed in the data is not present in the simulations. **A**: An unexpected partial cross-over is produced by the QLV model. It is caused by numerical errors, and more precisely by small (less that 0.2%) differences between the integral of the elongation speed and the change in muscle length. Such differences are not surprising since these simulations were carried out using the length measurements from the experiments. In the inset we plot the results of the same simulations after manually scaling the elongation speed so that its integral matches the change in muscle length. As expected, no cross-over occurs. In both cases, the traces for the fastest and slowest elongations only converge very late. **B**: No cross-over is observed in the AQLV simulations, and convergence occurs even later than with the QLV model.

When these same elongations are simulated using the QLV model (Fig. 7A), some cross-over between the three fastest traces is actually observed. This unexpected result is caused by small numerical errors. Because these simulations were carried out using the actual muscle length measured during the experiments, the integral of the elongation speed was not always identical to the change in muscle length. The difference was always less than 0.2%, but this was sufficient to produce the observed cross-over. In the inset in Fig. 7A we plot the results of the same simulations after manually scaling the elongation speed so that its integral exactly matches the change in muscle length. As expected, no cross-over occurs. Note that this artifactual cross-over is much less extensive than that observed in the data, where even the two slowest elongations were involved. The AQLV model (Fig. 7B) instead predicts no cross-over at all, in spite of the small numerical errors. For both models it takes a very long time for the fastest trace to converge with the slowest one.

### Toward a New Model

What we have shown so far forces us to conclude that both the QLV and the AQLV models cannot be used to predict the force generated by passive eye muscles under natural conditions. A new model is needed. Several methods can be used to identify a nonlinear viscoelastic model, but given that the QLV and AQLV models are capable of fitting step-wise elongations (admittedly with a fairly large number of parameters), we decided to modify these models to also fit the saccadic and constant-speed elongations. Ideally an acceptable fit would be achieved without adding too many parameters. To guide the design of such a model, a more quantitative analysis of the failure of the current models can be helpful. Accordingly, we defined as “purely viscoelastic force” the difference between the force measured (or predicted by a model) at the end of an elongation, and the elastic force predicted by the static length-tension relationship at that length. We then computed the ratio between the purely viscoelastic force measured and that predicted by the model. We call this measure the *Data/Model viscoelastic ratio*.

**Figure 8:**
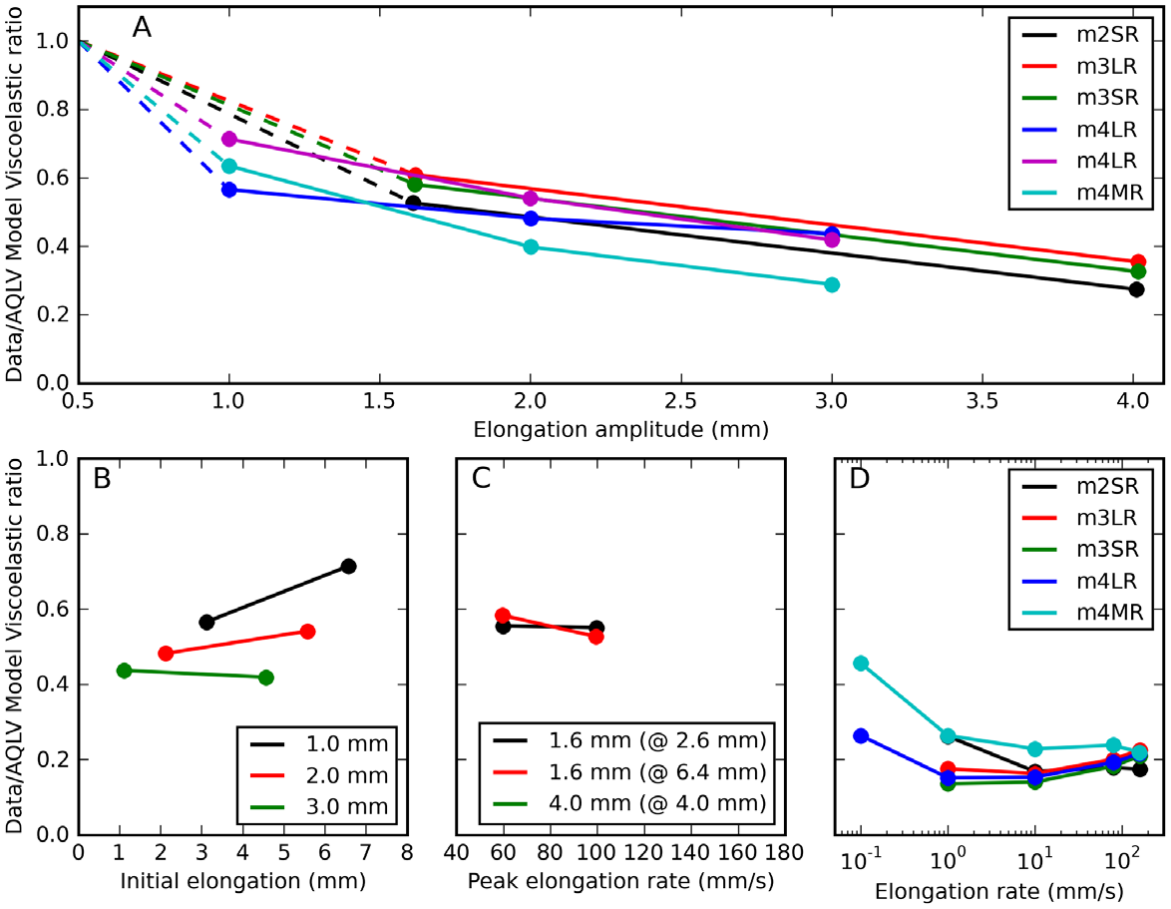
Data/model viscoelastic ratio. Ratio between the purely viscoelastic force measured at the end of an actual elongation, and the purely viscoelastic force predicted by the AQLV model at the same time. This is an index of how much the AQLV model overestimates the actual force, once purely elastic and viscous forces have been removed (see text for details). **A**: The index is plotted as a function of saccadic elongation amplitude, and each dot represents a different saccadic elongation. Saccadic elongations sharing the same final elongation (applied to the same muscle) have the same color and are joined by a solid line. The dashed lines indicate that this ratio should approach unity for 0.5 mm elongations (the length of our step-wise elongations; we never actually tested 0.5 mm saccadic elongations). **B**: The index is plotted as a function of initial elongation, for three different amplitudes. Black: 1 mm. Red: 2 mm. Green: 3 mm. Initial elongation affects the viscoelastic ratio in otherwise identical saccadic elongations. Data from lateral rectus in m4. **C**: Viscoelastic ratio as a function of peak elongation rate, for three different sets of saccadic elongations. Black: 1.6 mm starting from a short initial elongation. Red: 1.6 mm starting from a large initial elongation. Green: 4 mm starting from an intermediate initial elongation. Data from superior rectus in m2. **D**: Viscoelastic ratio as a function of elongation rate for constant-speed elongations.

We first looked at how this ratio varies as a function of elongation amplitude for saccadic elongations having different amplitudes but the same final length (such as those shown in Figs. 2 and 6), separately for each muscle. For the AQLV model we found (Fig. 8A) that in all cases this ratio decreases with the amplitude of the elongation, i.e., the AQLV model becomes progressively less accurate as the amplitude of a saccadic elongation increases. This finding is not surprising given the results reported in Fig. 4; however, this analysis also reveals that this ratio decreases less than proportionally with elongation amplitude. This is obvious if we posit (dashed lines) that this ratio is one for an amplitude of 0.5 mm (we did not actually induce saccadic elongations that short). This assumption rests on the observation (Fig. 4) that the model and the data agree remarkably well over the first 0.5 mm of a larger saccadic elongation, and that the model fits very well the force induced by 0.5 mm step-wise elongations. Even if we were to disregard this inferred data point, our observation is also supported by the (admittedly few) sets containing three saccades (cyan, blue and magenta lines). When we apply this analysis to the QLV model, we obtain the same result, but there is overall less attenuation, so that the lines are less steep and the lowest ratio is 0.5 (not shown).

Next we compared saccadic elongations having exactly the same amplitude and velocity, but starting from different initial lengths (three sets only, from one muscle). We found (Fig. 8B) that the relative discrepancy between the AQLV model and data is larger (i.e., the data/model viscoelastic ratio is lower) at small initial elongations (this is not necessarily true for the absolute model discrepancy, as the forces at play are higher at large lengths). This effect of initial elongation seems to decrease with the amplitude of the elongation, but because in these sets amplitude and initial elongation are correlated we cannot be sure that this is actually true. At any rate, it is clear that the viscoelastic ratio cannot simply be a functional of the elongation rate/amplitude. We also compared saccadic elongations starting from the same initial length, with the same amplitude, but with different peak velocities (Fig. 8C). As we noted above, the speed differences we used were too small to induce large changes in either the data or the simulations; this is reflected in the virtually constant viscoelastic ratios. Finally, we compared different constant-speed elongations (Fig. 8D), separately for each muscles tested (unfortunately the slowest speed of elongation was only used in the last monkey). In this case the discrepancy between model and data is always quite large, and the model appears to do better at very low speeds. In all these tests the QLV model behaves similarly (not shown).

### Attenuated Nonlinear Viscoelastic Model (ANLV)

From the above described data, it appears clear that in all cases the models overestimate the force generated by the muscle during extensive elongations, and increasingly so as the amplitude increases. Muscle behavior is thus reminiscent of the drop in viscous force observed in thixotropic materials: in structured liquids (e.g., gels, creams, paints, suspensions), externally imposed stresses and strains can induce reversible microstructural changes, which result in a temporarily reduced viscosity and possibly elasticity [14]. This process is often called *breakdown*. Once at rest, the microstructural changes are reversed, but the speed of this process (often called *rebuilding*) can vary widely across materials. The underlying cause of these phenomena has not been firmly established. However, it is commonly assumed that within thixotropic materials macromolecules spontaneously organize in a sort of network, whose junctions (entanglements) can be fairly easily destroyed by external actions, but also automatically reform at rest. Importantly, in a thixotropic material during the breakdown phase the viscosity decreases over time as a constant shear rate is imposed, eventually reaching a constant value. In contrast, in a shear thinning fluid viscosity is a function of the current shear rate, decreasing as the shear rate is increased.

The reduction in viscous force over time at a constant shear rate observed in thixotropic materials is analogous to the increasing discrepancy between the data and the predictions of our fixed-viscosity models observed during stretches. Also, the small differences observed when saccadic elongations of different speed but the same amplitude are applied (Fig. 8C), and to a large extent during constant-speed ramps (Fig. 8D), point to the elongation rate itself as not being particularly important. It would thus seem that the discrepancy between the predictions of the models and the forces recorded in muscle could be accounted for by an attenuation analogous to that observed in thixotropic materials. With this in mind, we adopt an equation often used [14], [15] in thixotropy research:
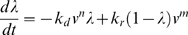
(3)In this equation, based on the so-called indirect microstructural approach, λ is a time-varying structural parameter: it is equal to one when the structure is completely built-up (maximum viscosity), and it becomes zero when it is completely broken-down (zero viscosity). The first addend on the right side measures how quickly the structure breaks down; the second term measures how quickly it builds back up. *v* is the shear rate (the speed of elongation in our experiments). *k_d_* and *n* control the break-down's speed; *k_r_* and *m* control the build-up's speed. Frequently *m* is equal to zero, and the build-up is thus only a function of time.

Because in our experiments we did not measure sequences of elongations (with the exception of some series of two elongations separated by small time intervals), we do not have any hard data to constrain the build-up side of the equation, and we thus drop it. Accordingly, we end up with the following, very simple, equation:
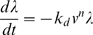
(4)where *v* is the elongation speed.

At this point we depart from the classic treatment of thixotropic materials, as we do not use λ to control a viscosity. Rather, we use λ to attenuate the purely viscoelastic component of the models. This is an important distinction, and we will address its implications at some length in the Discussion section.

For the QLV model (see Eq. 1) we then have:
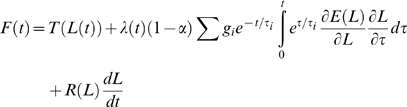
(5)and for the AQLV model (see Eq. 2) we have:
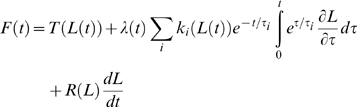
(6)


Of course it would be great if we could account for all the data by adding only two DOF (*k_d_* and n) to the original model. However, it is obvious that this simple formulation cannot possibly be sufficient. First of all, we saw that the initial phase (approximately 0.5 mm) of each smooth elongation is already fit by the models, without requiring any modification. However, according to Eq. 4, other things being equal λ drops faster at the beginning of an elongation (when λ is larger) than at the end. There is a simple way to get around this problem: we can initialize λ to a value larger than one, which we will indicate with λ_0_, and then use for the attenuation the smaller of λ and one. This adds another degree of freedom to the model.

Another limit of this model is that, since elongation speed is the only variable in Eq. 4, it cannot account for the different attenuation observed when we compare the model estimate and muscle recording for elongations with the same amplitude and speed, but starting from a different initial length (Fig. 8B). As noted above the attenuation decreases as length (and thus force) increases. Because this behavior appeared to be a nonlinear function of length, we guessed that it might be more or less linearly proportional to force (we do not have enough data to prove it either way). To account for this relationship, we thus made the break-down a function of the overall muscle force:
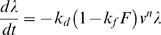
(7)This brings the number of DOF in this part of the model to four.

A third limit with this model is that it implicitly assumes that all of the viscoelastic processes are equally attenuated. That seems to be a pretty strong constraint, and in fact a detailed analysis of the data (not shown) rules it out. Rather than using a separate attenuation factor λ for each time constant, which would bring the number of DOF to 28 (7 by 4), we picked a different scaling factor γ_i_ for each time constant τ_i_. This is accomplished by multiplying the force prediction generated by each purely viscoelastic process in Eqs. 5 and 6 by a factor β_i_:
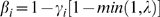
(8)Because we impose that the γ_i_ factors are bounded between zero and one, the gain factors β_i_ are also so bounded, and λ thus represents the maximum attenuation (i.e., lowest gain) across the viscoelastic processes. Because of this constraint, introducing the γ_i_ factors adds six, not seven, DOF, bringing the total to ten for the attenuation part of the model. The need to independently attenuate each viscoelastic process was the basis of our use of Eq. 1 to implement the QLV model, as opposed to its more usual formulation (Eq. 13 in our previous paper [4]).

Putting it all together, from the QLV model we derive:
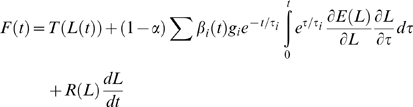
(9)and from the AQLV model we derive:
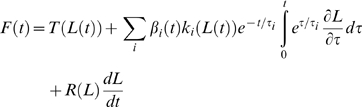
(10)Note that no attenuation is applied to either the asymptotic length-tension relationship or the purely viscous component. We refer to our model as the attenuated nonlinear viscoelastic model (ANLV); whether it is based on the QLV or on the AQLV model is conceptually irrelevant, and we thus do not adopt different names.

### System Identification

Now that we have derived a new set of models, with ten additional DOF, the next step is to use the forces we recorded in monkey EOMs to fit these additional DOF. Given the complexity of the fitness landscape, it is virtually impossible to achieve this using local optimization methods. We thus turned to a genetic algorithm. The optimization tool used the model to simulate quick-steps, saccadic elongations, and constant-speed elongations, looking for the set of parameters that yielded the best overall fit. When fitting this type of model to multiple data sets the choice of the error function to be minimized is crucial, since an incorrect choice can very easily push the algorithm to optimize one parameter at the expense of the others. To avoid falling into this trap, for each elongation the model-data error was computed as the sum of the mean squared error during the elongation phase plus a set of seven mean squared errors computed over seven intervals of the relaxation phase (for each time constant in the model, we picked the interval that started with the end of the elongation and lasted three times the time constant). Note that these are mean squared errors, so that the duration or number of data points in each interval becomes irrelevant; a simple sum of squared errors would strongly favor the slowest processes. Finally, the errors for each elongation tested are summed together. The algorithm ran through 50 generations, with a population size of 75 designs. The seed population was determined using a Sobol DOE algorithm. The best overall solution (i.e., the one with the lowest error) was then selected. This procedure was repeated for each muscle, and separately for the QLV and the AQLV based models. In Table 1 we report the values of the parameters that were found following this procedure for the QLV-based model. In Table 2 the values for the AQLV-based model are listed.

**Table 1:** Parameters for the ANLV model based on the QLV model.

	m2SR	m3LR	m3SR	m4LR
***k_d_***	0.200	0.240	0.310	0.320
***n***	1.490	1.410	1.450	1.290
**λ_0_**	3.000	2.060	3.000	1.930
***k_f_***	0.001	0.001	0.001	0.001
**γ_1_**	0.980	0.000	0.000	0.000
**γ_2_**	0.110	0.000	0.000	0.000
**γ_3_**	0.810	0.020	0.000	0.180
**γ_4_**	0.210	0.680	0.580	0.730
**γ_5_**	0.860	0.850	0.630	0.530
**γ_6_**	0.300	0.240	0.470	0.580
**γ_7_**	0.370	0.660	0.760	0.830

**Table 2:** Parameters for the ANLV model based on the AQLV model.

	m2SR	m3LR	m3SR	m4LR
***k_d_***	0.270	0.280	0.600	0.450
***n***	1.260	1.210	1.070	1.180
**λ_0_**	1.620	1.410	1.510	1.640
***k_f_***	0.001	0.001	0.001	0.006
**γ_1_**	0.270	0.000	0.200	0.000
**γ_2_**	0.040	0.000	0.000	0.000
**γ_3_**	0.740	0.420	0.420	0.510
**γ_4_**	0.800	0.990	0.850	0.990
**γ_5_**	0.860	0.670	1.000	0.740
**γ_6_**	0.990	0.950	0.960	0.860
**γ_7_**	0.880	0.980	0.880	1.000

### New Models Performance

The attenuation introduced in the ANLV model rectified, at least qualitatively, all the failures of the models it is based on. For the QLV model the overall error was reduced to between 1/20^th^ and 1/100^th^ of the original error. For the AQLV model the overall error was reduced to between 1/1000^th^ and 1/2000^th^ of the original error (which of course was much larger than for the QLV model). In all cases the model based on the AQLV model ended up providing a slightly better fit, but for all practical purposes the final models are equally good.

**Figure 9:**
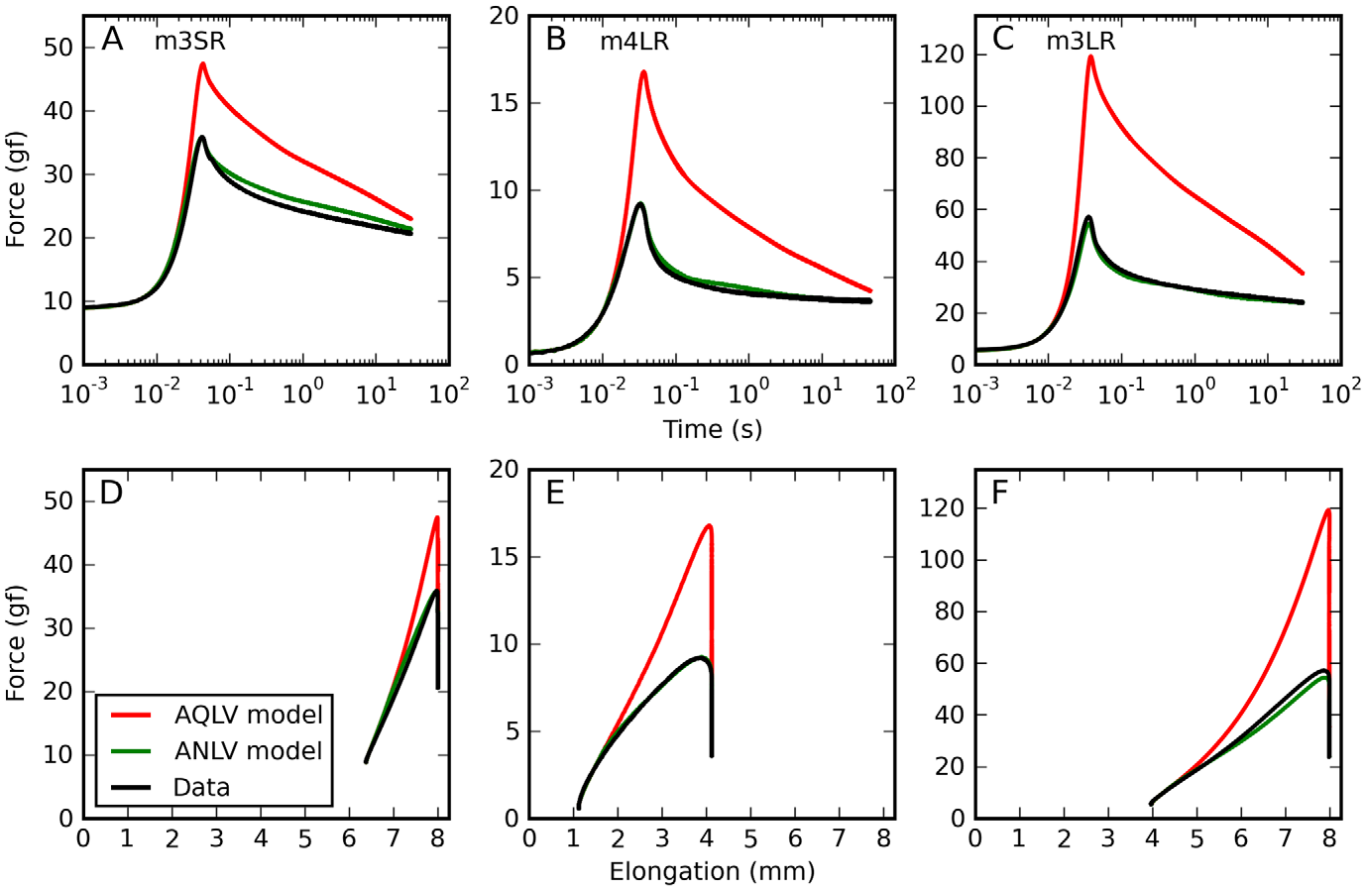
Saccadic elongations: ANLV predictions. Forces induced by saccadic elongations (same elongations shown in Fig. 4). Data (black), force predicted by the AQLV model (red) and force predicted by the ANLV model based on the AQLV model (green). **A**: Short elongation (1.6 mm) starting from a large initial elongation, applied to the superior rectus in m3. **B**: Intermediate elongation (3.0 mm) starting from a small initial elongation, applied to the lateral rectus in m4. **C**: Large elongation (4.0 mm) starting from an intermediate initial elongation, applied to the lateral rectus in m3. **D–F**: Here we show, for the same elongations represented in the top row, the force induced in the muscle as a function of length. Whereas the AQLV model fits the data well only for the first 0.5 mm of the elongations, the ANLV provides a good fit throughout.

In Fig. 9 we plot the same elongations shown in Fig. 4; the ANLV model prediction (based on the AQLV model) is shown in green. The improvement over the AQLV model is obviously significant, and it is difficult to expect anything better since the parameters of the models where not optimized for these individual traces, but over a large number of elongations in each muscle. The ANLV extension of the QLV model reproduces these traces almost as well, with only a slight overestimation in the second trace during the first 3 s of the relaxation phase.

**Figure 10:**
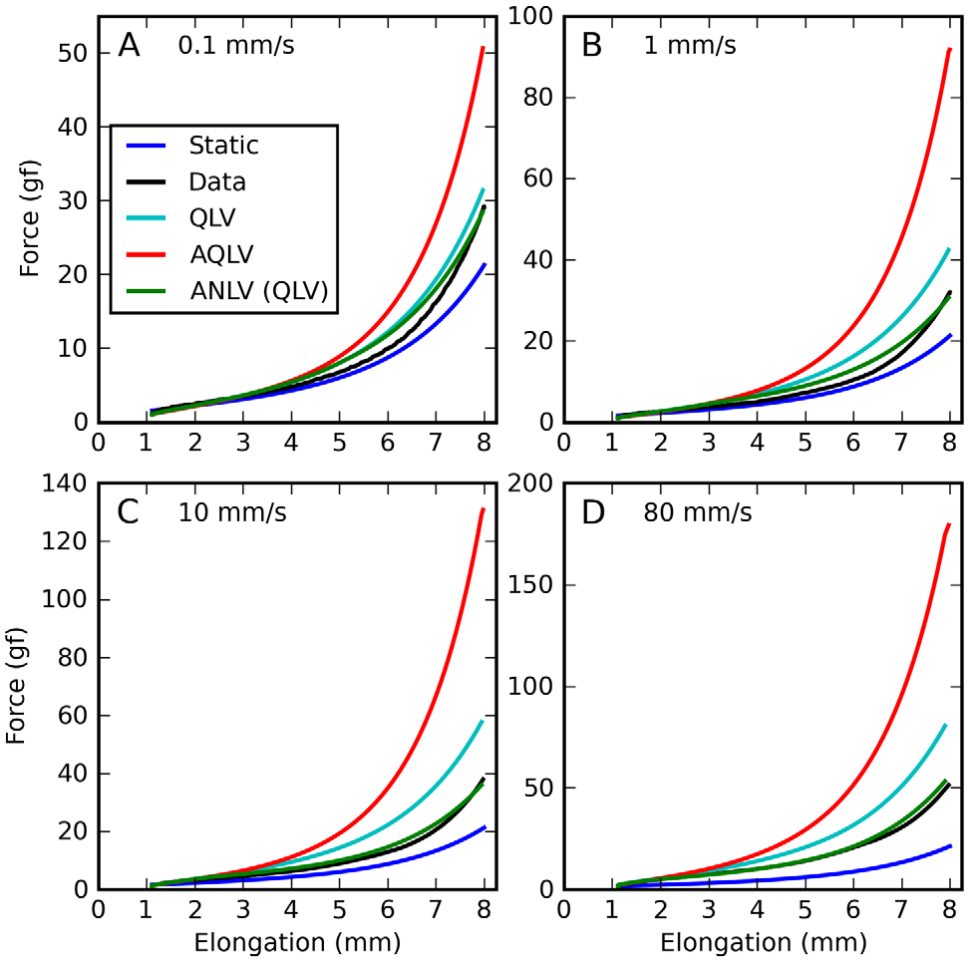
Constant-speed elongations: ANLV predictions. Blue: Static length-tension relationship. Black: Force measured in muscle. Cyan: force predicted by the QLV model. Red: force predicted by the AQLV model. Green: ANLV (based on the QLV) model prediction. **A**: Stretch at 0.1 mm/s. **B**: Stretch at 1 mm/s. **C**: Stretch at 10 mm/s. **D**: Stretch at 80 mm/s. In all cases the ANLV model predicts the generated force quite well, vastly outperforming the AQLV model. Note very different ordinate scales.

In Fig. 10 we show the force predicted during a set of constant-speed ramps, as we did in Fig. 5 for the original models. Here we chose a different format for the figure to make it easier to compare the model predictions to the data. The ANLV model produces virtually identical simulations regardless of whether based on the QLV or the AQLV model, and so only one of them is shown. The improvement is again very obvious, even though there is still a bit of force overestimation between 5 and 7 mm of elongation, and a slight undershoot at the final length.

**Figure 11:**
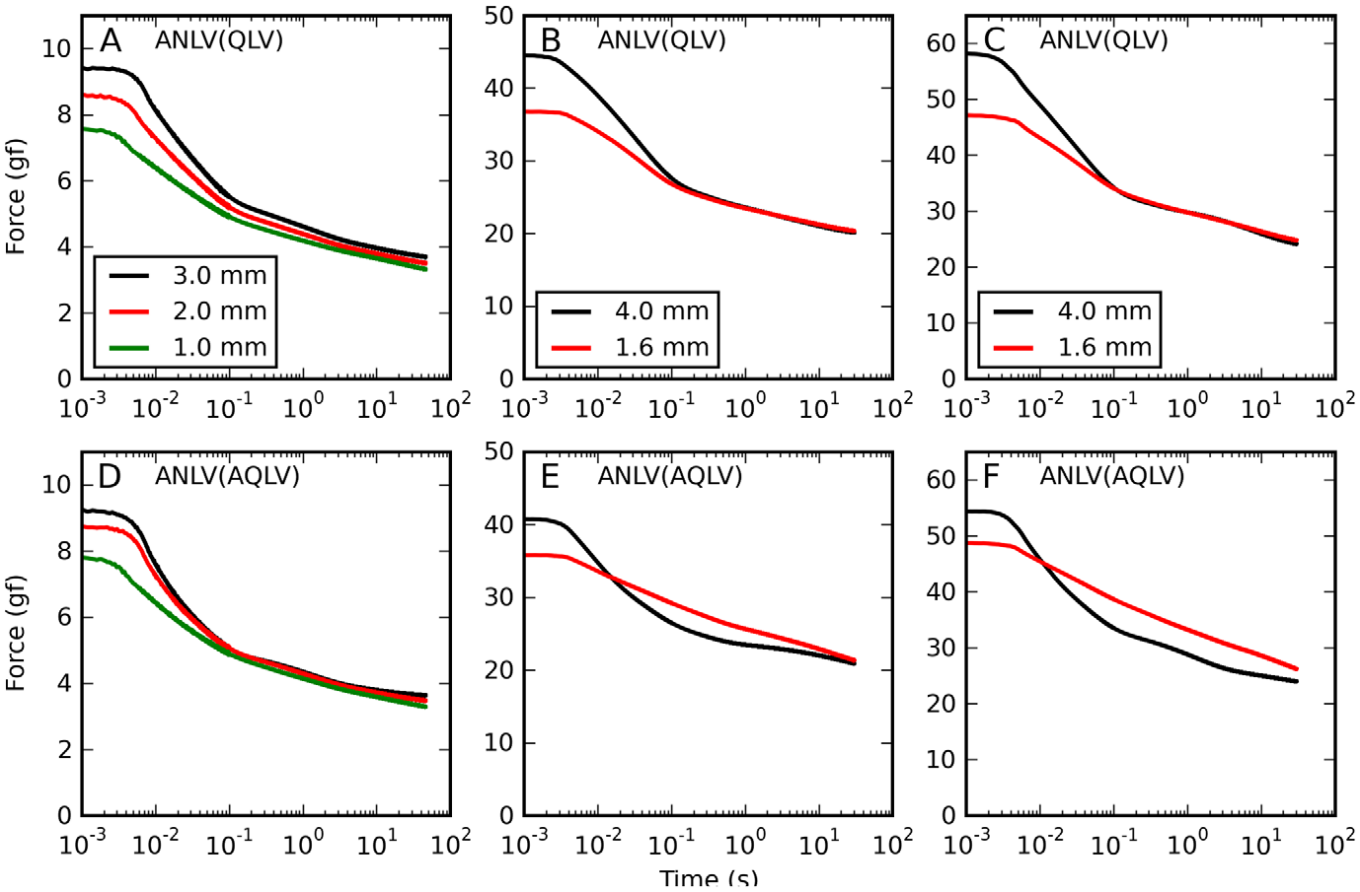
Force relaxation after saccadic elongations: ANLV predictions. Simulations of the same elongations described in Figs. 2 and 6 using the ANLV model. In each panel in the top row we plot simulations of elongations having different amplitudes but the same final length, all done with the ANLV model based on the QLV model. In the bottom row simulations of the same elongations with the ANLV model based on the AQLV model are shown. In all cases the ANLV model performs better than either the QLV and the AQLV model. However, in this case the model based on the QLV model does a better job, as the simulations in B & C match the data much better than those in E & F. More precisely, the cross-over observed in E and F is considerably larger than the slight one observed in the data (Fig. 2).

In Fig. 11 we report simulations of both ANLV models for the same elongations that were simulated with the QLV model in Fig. 6 (cf. data in Fig. 2). Again, here we are comparing saccadic elongations characterized by different amplitudes but a common final elongation. The improvement is again clear; the ANLV model based on the QLV captures particularly well the two aspects of the data that were not captured by the previous models, namely the traces coming together within about 100 ms from the end of the elongation (Fig. 11A), and a slight cross-over at larger final elongations (Fig. 11B–C). The ANLV model based on the AQLV does also considerably better than previous models, but it does not match the data as well, as the cross-over in Fig. 11E&F is considerably more extensive, and it is mostly due to an under-attenuation of the shorter movement (red trace).

**Figure 12:**
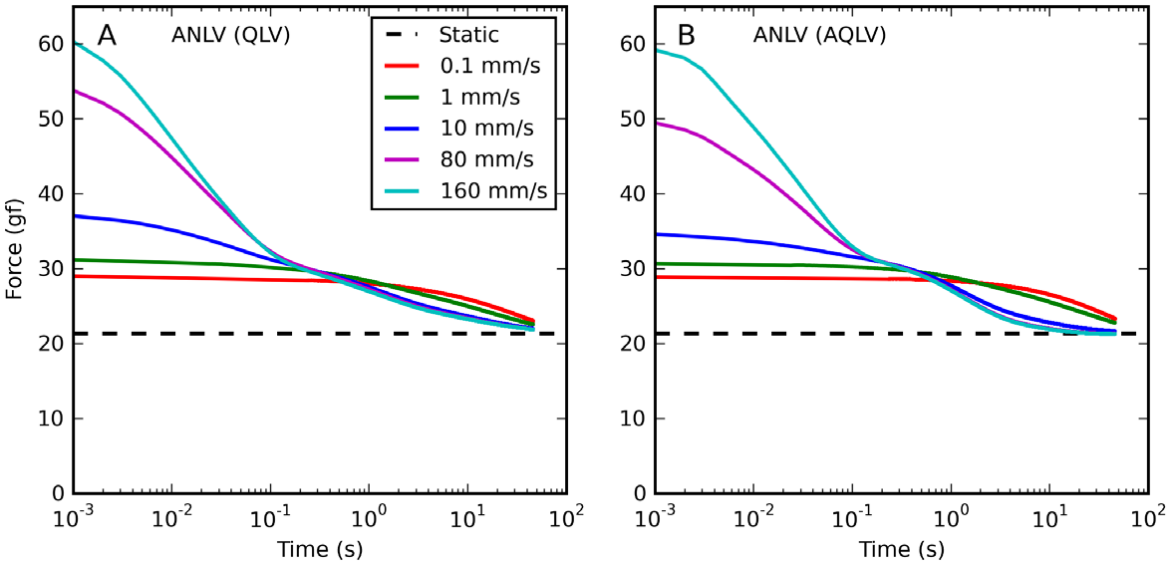
Force relaxation after constant-speed elongations: ANLV predictions. Simulations of the same elongations described in Fig. 7 using the ANLV models. The cross-over of the traces observed in the data (Fig. 3) is captured by the ANLV model, whether based on the QLV or AQLV model. In the data this cross-over is more orderly than in the model, and the model does not perfectly capture the peak force, but this is nonetheless quite an improvement over previous models (compare with Fig. 7).

To complete this comparison of the ANLV model with the two models that we described above, in Fig. 12 we simulate the relaxation response following constant-speed elongations. Here we show the results obtained with both versions of the ANLV model, and they should be compared to the data reported in Fig. 3A. Obviously it is not a perfect match, but the magnitude of the forces and the crossing-over of the traces are well captured. The improvement over the previous models (Fig. 7) is considerable, both quantitatively and qualitatively.

So far all the comparisons between data and model simulations have been based on a model that was fit to the data (albeit to a large data set, not to individual elongations). Needless to say, any model so built cannot be considered anything more than a compact description of the data. To verify whether our model actually has any predictive power, we also checked how the model behaves when it is applied to elongations that were not part of the optimization set. Rather than excluding individual saccadic elongations or constant-speed stretches from the optimization set, as is usually done in cross-validation, we tested the model on two completely different paradigms: sequences of two step-wise elongations (0.5 mm each), and sequences of two saccadic elongations (1.6 mm each). Note that neither the sequences nor the individual elongations making up each sequence were part of the training set, and thus are *bona fide* predictions of the model.

**Figure 13:**
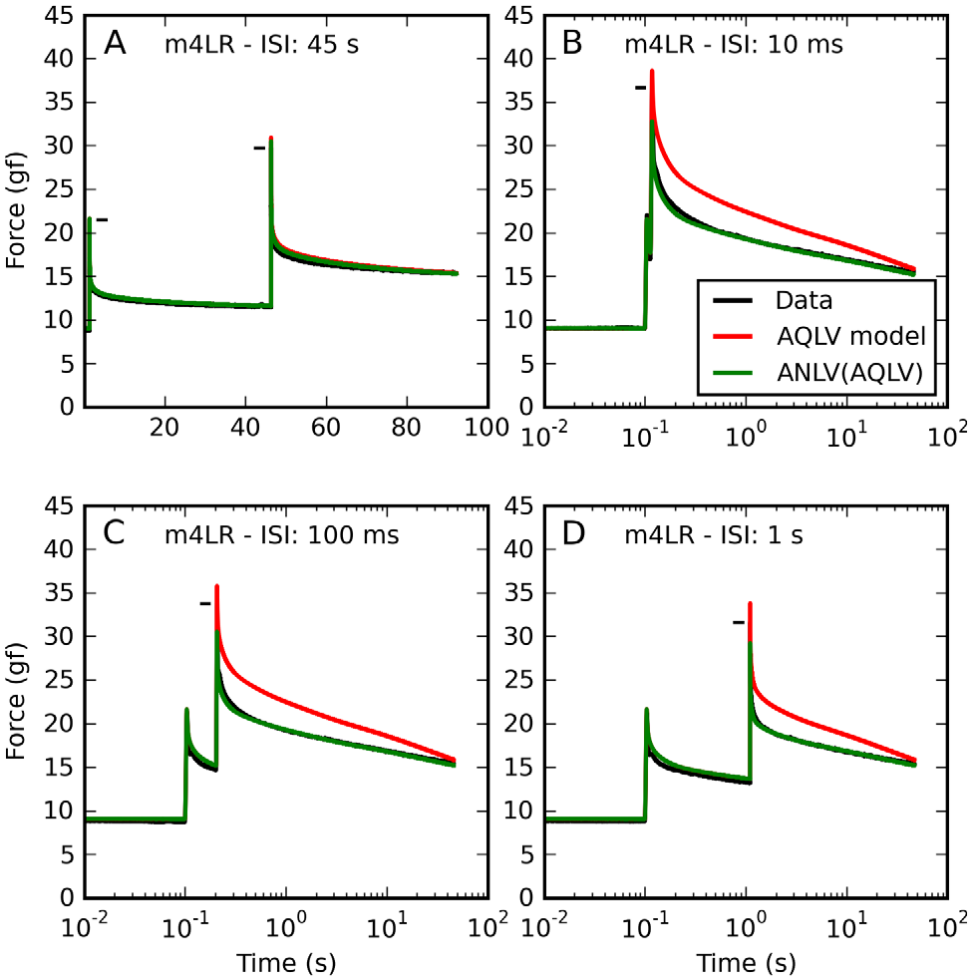
Double-steps: Data and ANLV predictions. Prediction of the force induced by sequences of step-wise (0.5 mm) elongations. Black: Force measured in muscle. Red: force predicted by the AQLV model. Green: ANLV model prediction. In each panel a different time separation (ISI) is shown. **A**: Here the ISI is 45 s, and the attenuation is reset before the second step. There is thus virtually no difference between the AQLV and the ANLV model, and they both reproduce the data quite well. **B**: Same as in A, but with a 10 ms ISI. There is no difference between the models after the first elongation, but the ANLV model is considerably better at predicting the force after the second elongation. **C**: Same as in B, but with a 100 ms ISI. **D**: Same as in C, but with a 1 s ISI. For clarity, the maximum force recorded is indicated using a small horizontal black bar.

In Fig. 13 we plot the double-step data. Each panel represents a different inter-step interval (ISI). At the largest ISI (panel A) the AQLV model (red line) and the ANLV model based on it (green line) are essentially identical, and reproduce the measured force (black line) well. The response of the two models is identical because the attenuation variable λ was reset to its initial value λ_0_ before the second step, and by design there was no attenuation during a 0.5 mm step (that was why we introduced λ_0_). At shorter ISIs both models predict the force induced by the first step; however, the force elicited by the second step is grossly overestimated by the AQLV model. We described this phenomenon at length in the previous paper in this series [4]. The ANLV model instead does quite a good job at predicting the force, especially during the decay (note that the peak force is somewhat underestimated, though). The ANLV model based on the QLV model does also a fairly good job, but not as good: the second step in the series is usually over-attenuated by approximately 1 gf (not shown).

**Figure 14:**
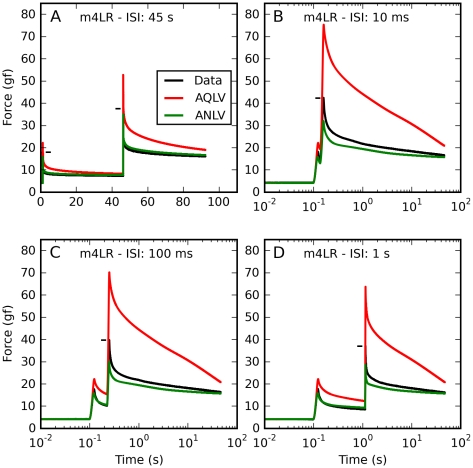
Double-saccades: Data and ANLV predictions. Prediction of the force induced by sequences of saccadic elongations (1.6 mm each). Black: Force measured in muscle. Red: force predicted by the AQLV model. Green: ANLV model prediction. In each panel a different time separation (ISI) is shown. **A**: Here the ISI is 45 s, and the attenuation is reset before the second elongation. Not surprisingly, the AQLV does a very poor job of fitting both elongations. Even the ANLV model is not perfect, overestimating the force during the second elongation. **B**: Same as in A, but with a 10 ms ISI. Again, the ANLV model is much better at predicting the force after the second elongation than the AQLV model, but it now underestimates the force. **C**: Same as in B, but with a 100 ms ISI. **D**: Same as in C, but with a 1 s ISI. (Note: these saccadic elongations were not used to fit the ANLV model, and are thus genuine predictions).

In Fig. 14 we plot the double-saccade data, again with different ISIs in each panel. In this case the AQLV model does a very poor job of predicting the force induced by either saccadic elongation, regardless of ISI. This is not surprising. The ANLV model does a fair job of estimating the force induced by either saccade at the largest ISI (again, λ was reset to its initial value λ_0_ before the second step). However, at shorter ISI the model invariably underestimated the force induced by the second elongation. The ANLV model based on the QLV model behaved similarly (not shown). We do not have any explanation for this failure; we can only speculate that maybe there is some recovery from the attenuation during the ISI (i.e., some rebuilding occurs during the ISI), which we have not modeled. Additional data will be needed to clarify this issue.

## Discussion

### Force during Natural Elongations

In the set of experiments described here we measured forces under elongations that mimic those experienced by muscles during saccadic eye movements, the most common kind of eye movement (performed about three times per second). In addition, we imposed constant-speed elongations. These were only partially physiologic, because smooth movement are not usually performed at very high speeds (our 10 mm/s elongation is the fastest that could be considered physiologic) and do not usually cover the entire oculomotor range.

In general, we found that these forces are significantly lower than those predicted by standard viscoelastic models fitted to small elongation steps. Importantly, we noted that during the elongation phase the forces positively correlated with the speed and amplitude of the elongation; however, during the post-elongation relaxation phase these differences tended to die down quite quickly, so that the major determinant of the force became the final muscle length. This finding is reminiscent of what we previously reported [4] about the forces induced by sequences of two elongations.

Taken together these two findings could have important implications for the oculomotor neural controller. They seem to suggest that the brain might not need to “compute” the passive force generated by a muscle following an eye movement, which would require some sort of internal model of the muscle itself, driven by an efference copy of the motor commands. Instead, it could learn to simply associate a final eye position with a post-elongation force template, or, more specifically, it could learn to simply generate a patterned motor output that is a function of the post-movement position of the eye. Strong experimental evidence indicates that, at least in primates, the brain is indeed capable of generating such a motor output, and that the post-movement eye drift (which is exactly what would ensue if the passive forces we described were not compensated) is a sufficient driver [16], [17].

At this stage this must be considered a speculation, since we have not collected enough data to make a stronger statement. Importantly, at the end of our constant-speed ramps we observed a systematic cross-over of the force traces, so that faster elongations result in higher peak forces, but lower relaxation forces. In one case (Fig. 3B) the force difference between the fastest and slowest trace ended up being a few grams force, certainly enough to induce eye drift. Because the forces do not even seem to converge to the same value, this raises the possibility that a true static length-tension relationship might not even exist. We derived [3] this relationship by extrapolating the asymptotic force following small and fast step-wise elongations, and all the other fast elongations that we applied did in fact relax to this curve. However, this is not enough to rule out that slower elongations might relax to higher forces, as is suggested by the decay observed after slow constant-speed elongations. Internal static friction (also referred to as stiction) could, for example, have such an effect. While this possibility is intriguing, it should be noticed that the cross-over always occurred at the limit of our stretching range (this was the case also in the two saccadic traces show in Fig. 2B&C), where the forces are highest. It is thus entirely possible that the cross-over between traces is a phenomenon that occurs only when the forces at play are very large, and might occur never, or extremely rarely, in physiologic conditions. Unfortunately, we did not test constant-speed elongations that terminated well before the limit of our stretching range. Further experimental work is thus necessary.

### Limits of Our Experimental Approach

As we noted in the preceding papers in this series, our *in vivo* preparation imposed several experimental constraints. Importantly, since some of the muscles that we pulled on were partially wrapped around the eyeball, the elongation of the passive muscle could be somewhat smaller than the motion of its tendon. Unfortunately, there was no feasible way to verify whether or how the posterior pole of the eyeball was deformed. Strictly speaking, the force we report here is thus the force that would be applied on the eyeball by a passive antagonist muscle when it is extended by the action on the globe of a shortening agonist muscle.

Nonetheless, it has hard to see how this could have affected *qualitatively* the findings here reported. In particular, it would be very hard to explain how the force after a large elongation is higher at the end of the elongation, but then becomes lower during the relaxation phase. Importantly, this result has been observed also, and possibly even more clearly, in a medial rectus muscle (cf. Fig. 3B), which does not wrap around the globe at all, and thus cannot induce translations or deformations of the eyeball.

Other experiments will be needed to quantify how the presence of the globe affects the forces generated in eye muscles.

### Comparison with Other Studies of Biological Materials

Rheological studies of biological materials have traditionally relied on simpler elongation patterns than those used here, with fast steps and small vibrations being the most widely used. There are however exceptions. First of all, it is commonly reported that biological tissues are strain-rate insensitive, meaning that the force induced by large elongations (like our constant-speed elongations) depends only weakly on the strain-rate. It was actually this observation that led Fung [6] to propose Neubert's continuous relaxation spectrum [18] as the reference implementation for the reduced relaxation function in the QLV model. Obviously, this is not what we found (cf. Fig. 2). It should however be noted that the strain rates usually tested are much lower than those used here. It is entirely possible that if we had restricted our range of rates from, say, 0.001 mm/s to 1 mm/s we would have also observed an approximate rate independence.

An exception to this common observation comes from a study on aortic valve tissue [19]. Here the authors used the QLV model with Neubert's continuous spectrum relaxation function to fit the data, separately for each strain rate. They found that indeed the strain rate affects the parameters of the model, but in essentially the opposite direction reported here, leading them to propose a *shear-thickening* effect. In other words, in their experiment following a constant-speed elongation the force dropped faster after slow elongations than after fast elongations (relative to the prediction of the QLV model). We have no explanation for this difference, other than that the tissues under examination are considerably different.

In another study on reconstituted collagen [20] the authors measured the force induced by small step-wise elongations followed by a hold period, and by slower elongations of the same amplitude followed by a return to the initial length. It was found that a standard viscoelastic model could fit all the data. However, the problems that we had in fitting the data with a standard model were contingent upon testing large elongations, which were not part of that study.

A phenomenon similar to the one described here has been recently reported in contractile fibroblasts [21]. Nekouzadeh and colleagues measured the force induced in contractile fibroblasts embedded in reconstituted collagen when they are subjected to large constant-speed elongations. They noted that during the post-elongation relaxation phase the force dropped faster after a fast stretch (equivalent to about 200 mm/s in our experiment) than after a slow stretch (equivalent to about 2.5 mm/s in our experiment). They determined that this force shedding is due to the depolymerization of the actin cytoskeleton; they suggested that this mechanism might be self-protecting, releasing mechanical stress. It should be noted however that in their preparation higher stretch rates did not yield higher peak forces, unlike what we found in EOMs.

### The QLV/AQLV Models for Eye Muscles

The identification of a nonlinear dynamic model from experimental observations is one of the most challenging system identification problems. Unless the model structure is known, so that only a small set of parameters needs to be estimated, acquiring the necessary data is usually a monumental task. Furthermore, the type of experiments to be carried out and the model formulation are heavily intertwined, so that one essentially determines the other. Fung's quasi-linear theory [5], [6], proposed almost 40 years ago, has represented the most successful framework to study nonlinear viscoelastic behavior. It has been used to model the mechanical behavior of countless viscoelastic materials.

In the previous paper in this series [4] we showed that the AQLV model proposed by Nekouzadeh and colleagues [7], and to a lesser extent Fung's original QLV model, can be used to reproduce the forces induced in passive muscles by small step-wise elongations. However, they fail to reproduce the force induced by sequences of two steps. As we explained, this failure was not just quantitative, but rather structural.

That failure was however not necessarily catastrophic. In many applications what matters most for these types of models is whether they are capable of predicting the forces likely to be experienced in physiologic conditions. For eye muscles this requires testing smooth elongations considerably larger than the steps previously used. Since Pipkin and Rogers [9] argued that in man-made materials the extrapolation from small steps to smooth elongations is quite straightforward, we looked forward to the possibility that at least one of the models might in fact behave quite well.

Our measurements of the force generated during these “natural” elongations indicate that it is consistently, and often egregiously, overestimated by previous models. This proves conclusively that the QLV theory fails to capture some fundamental property of passive eye muscles, and consequently cannot be used to model them. In spite of their large number of parameters, these models simply cannot reproduce the behavior of extra-ocular muscles under the wide range of conditions that are encountered in everyday life.

One point that needs to be stressed is that this failure occurs not only at the extreme edges of the oculomotor range, but involves also the central region where the eyes spend most of their time. In our first paper [3] we had shown that in this region (up to approximately 3 mm of elongation, corresponding to 18° of eye rotation) the static length-tension relationship was essentially linear. Since in the QLV model the elastic response (i.e., the nonlinear component of the model) is directly proportional to the static length-tension relationship [4], this implies that our QLV implementation is essentially a linear model in this range. In our previous paper we had already shown that this model failed to predict the response of sequences of two steps, thus ruling out the ability of a linear model to approximate an eye muscle, even in this range. What we have shown here further reinforces this conclusion. While unfortunately we have not measured saccadic elongations that are wholly contained in this range, it is easy to ascertain from Fig. 4 (panels B and E) that the QLV model is incapable of reproducing movements larger than those used to fit its parameters, even in this range (note in particular the large deviation between the blue and black traces at the 3 mm elongation point in Fig. 4E). To summarize, if a linear model were fit to small elongations (say equivalent to 3° saccades) it would grossly overestimate the passive force generated during larger elongations; if instead it were fit to large elongations, it would grossly underestimate the passive force generated during smaller elongations.

After our previous paper was accepted for publication, another study dealing with the viscoelasticity of EOMs was published [22]. The conclusion of that study was that the QLV model is able to reproduce the mechanical properties of eye muscles, the opposite of what we have concluded. The apparently contradictory conclusions drawn in our and their study can be resolved quite easily. First of all, their preparation was quite different (they used post-mortem sections of bovine EOMs). Second, and most important, Yoo and colleagues applied to their specimens only the classic fast step-wise elongations and slow (equivalent to less than 0.5 mm/s in our experiment) cyclical elongations. Had we applied their criterion to our fast-step data, we would also have concluded that the QLV model was appropriate. We did not, and argued instead in favor of the AQLV model, because we used a stricter criterion. It could be argued that our criterion was too strict, but it was in line with the criteria used in other studies that rejected the QLV model. Our strongest rationale for rejecting the QLV theory came however from sequences of two elongations, and from the large smooth elongations described in this paper; neither of these were part of their study. Our studies are thus not contradictory. In some respects they are actually complementary, since their preparation allowed them to investigate the behavior of EOMs over larger time scales (they measured the relaxation response over 1500 s).

### The ANLV Model

One of the goals of our experimental inquiry was to identify a model of the passive eye muscle that we could then incorporate in our model of the eye plant [23], [24]. The model would not need to be particularly elegant or insightful, but would have to generate reasonable predictions. We had hoped that this could have been accomplished by simply finding the appropriate parameter values for an existing model, but this was not possible. Of course, proving a model wrong is an important accomplishment in itself [25], but we felt that not providing an alternative would have been unsatisfactory on many levels.

Unfortunately, coming up with an entirely new model of a highly nonlinear system is not an easy endeavor. Furthermore, our experiments were designed with the QLV theory in mind, and a different type of model might very well require a different set of experiments for proper identification. Accordingly, we looked for an enhancement to the existing models that would be parsimonious and would yield a reasonably good model of passive eye muscles. The solution we found, inspired by studies of thixotropic materials, meets both criteria. Because the failure of the previous model tested boiled down to an overestimation of the force measured in muscles during large, smooth, elongations, it seemed natural to us to retrofit that model with a variable attenuator. A careful analysis of the data revealed that the attenuation had to be a functional of length and speed. Also, the various processes that are part of the QLV model, each characterized by a different time constant, needed to be attenuated somewhat differently. It quickly became clear to us that, with some small modifications, the structural parameter used in thixotropy research behaves a lot like the attenuation parameter that we needed. This observation led to our ten DOF extension of the QLV/AQLV models, which we called the attenuated nonlinear viscoelastic model (ANLV). This model is conceptually similar to models of viscoelastic thixotropic materials, the most influential being the one proposed by Acierno and colleagues in 1976 [26].

We have shown here that the ANLV model, while far from perfect, represents a significant improvement over the currently available models, and might be used with a fair amount of confidence in simulating the response of passive extraocular muscles to arbitrary elongations. Our attempt at predicting the outcome of experiments that were not used to fit the model was only partly successful. The double-step data was fit remarkably well, better than we expected. Our predictions of the force induced by sequences of two saccadic elongations, which of course is much more interesting as it mimics more physiologic conditions, were instead only marginal. While previous models predicted forces much larger than those observed, the ANLV model underestimated the forces. Since our attenuation function did not incorporate a recovery term, it is possible that the lack of this process is to be blamed for this failure. Because the data collected does not allow us to constrain this putative process, more experiments are necessary. One point that must be remarked, though, is that implementing such a recovery process is not trivial, and certainly cannot be accomplished by simply introducing in Eq. 4 the build-up term that we dropped from Eq. 3. In thixotropic research, the structural parameter λ scales the viscosity of the material; in contrast, in the ANLV model λ scales the purely viscoelastic force (i.e., both the viscosity and the stiffness, so that the time constant does not change). Thus, if this scaling parameter were to change during the decay phase after an elongation, the decay force would no longer decay exponentially, and it could actually become non-monotonic, a phenomenon that we never observed.

We want to stress that we make no claim that this model has any physiologic basis. Consequently, we have made no attempt to interpret the values of its parameters, or to compare them across muscles. The only observation that we'd like to make is that the following pattern seems to hold: processes associated with long time constants are attenuated more than those associated with short time constants. This is reminiscent of the model of polymer melts by Acierno et al. [26], in which a continuous relaxation spectrum is progressively truncated under steady shearing. We believe that this can be rationalized in the following way: in a viscoelastic model, during an extended elongation the force builds up much more in elements that are associated with a long time constant, since each element can be seen as a leaky integrator of the elongation rate, with shorter time constants indicating larger leaks. If all processes can only “support” a certain force, processes with longer time constants will then need to be attenuated more. To make an analogy based on the theory of structured fluids, we could then visualize our model as a network of macromolecules, in which different sub-networks exhibit different viscoelastic behaviors, but in which the entanglements break with a probability that is mostly a function of the stress to which they are subjected.
